# A method for efficient Bayesian optimization of self-assembly systems from scattering data

**DOI:** 10.1186/s12918-018-0592-8

**Published:** 2018-06-08

**Authors:** Marcus Thomas, Russell Schwartz

**Affiliations:** 1 Computational Biology Department, Carnegie Mellon University, 5000 Forbes Ave, Pittsburgh, USA; 2 Department of Biological Sciences, Carnegie Mellon University, 4400 Fifth Avenue, Pittsburgh, USA

**Keywords:** Gaussian process regression, Kernel learning, Bayesian optimization, Small-angle scattering, Molecular self-assembly, Stochastic simulation, Rule-based modeling

## Abstract

**Background:**

The ability of collections of molecules to spontaneously assemble into large functional complexes is central to all cellular processes. Using the viral capsid as a model system for complicated macro-molecular assembly, we develop methods for probing fine details of the process by learning kinetic rate parameters consistent with experimental measures of assembly. We have previously shown that local rule based stochastic simulation methods in conjunction with bulk indirect experimental data can meaningfully constrain the space of possible assembly trajectories and allow inference of experimentally unobservable features of the real system.

**Results:**

In the present work, we introduce a new Bayesian optimization framework using multi-Gaussian process model regression. We also extend our prior work to encompass small-angle X-ray/neutron scattering (SAXS/SANS) as a possibly richer experimental data source than the previously used static light scattering (SLS). Method validation is based on synthetic experiments generated using protein data bank (PDB) structures of cowpea chlorotic mottle virus. We also apply the same approach to computationally cheaper differential equation based simulation models.

**Conclusions:**

We present a flexible approach for the global optimization of computationally costly objective functions associated with dynamic, multidimensional models. When applied to the stochastic viral capsid system, our method outperforms a current state of the art black box solver tailored for use with noisy objectives. Our approach also has wide applicability to general stochastic optimization problems.

## Background

As efforts to build predictive quantitative models of complex systems in biology have grown increasingly complex and comprehensive (e.g., [1, 2]), they have inevitably had to deal with the challenge of capturing molecular self-assembly chemistry. Self-assembly chemistry is an essential part of nearly every important function of a living cell, yet has long proven exceptionally challenging due to their large sizes, long time scales, and explosive pathways spaces. Simulations can provide a way to examine details of assembly unavailable to direct experimental observation, but are computationally demanding for complex assemblies, leading to a body of specialized simulation methods specifically for simulating molecular self-assembly chemistry [3–12]. Furthermore, learning parameters needed by these simulations is itself a very difficult problem for assembly systems, likewise requiring specialized methods [13, 14]. See [15] for a recent review.

We previously showed that it is possible to simulate realistic scales and parameter ranges of complex self-assembly reactions, with specific focus on virus capsid assembly as a model system, by using coarse-grained, rule-based models [4]. These rule-based models were originally implemented via Brownian particle models [4, 5, 8, 11] and later via fast stochastic sampling algorithms [7, 9, 10], approaches that have since seen widespread use in modeling capsids and other complex reaction systems. Accurately parameterizing such models from experimental data, though, remains challenging. Standard methods for model fitting in biochemistry, particularly the Bayesian model-inference methods that have become the favored approach in the field [16], are unusable for non-trivial self-assembly systems due to their exceptionally high computational cost, large pathway space, and inherent stochasticity [15]. In past work, we showed that it was possible to learn detailed quantitative parameters of these models via simulation-based model fitting to static light scattering (SLS) measurements of bulk assembly in vitro [13, 17], primarily by bringing to bear specialized optimization techniques from the field of Derivative-Free Optimization (DFO) [18]. Together, these contributions made it possible to infer the subunit-level pathway space of real capsids assembling in vitro, which in turn can be applied to explore how pathway usage might differ under more realistic models of the intracellular environment [19, 20]. The reliability of such inferences is uncertain, however, due to limits of the data in precisely and uniquely identifying a specific model and the difficulty of accounting for model uncertainty with these classes of methods. The present work focuses on improving parameter fitting methods in terms of potential experimental techniques to which one can fit models and parameter inference algorithms that can be applied for the fitting.

Computationally, we seek to bring to self-assembly the advantages of Bayesian model inference in exploring the space of possible solutions. We approach the problem of quantifying uncertainty in parameter estimation by constructing a probabilistic model of the objective function using Gaussian process (GP) models [21]. This GP method is a variant of a technique called kriging [22] that has previously proven valuable in other contexts for solving computationally demanding model inference problems under uncertainty. GP models are defined by mean and covariance (kernel) functions, and specify a prior on the space of possible functions. As simulations at successive parameter values are completed, the prior is updated, forming the posterior which is used in prediction. New data points for sampling are selected based on the current properties of the process and user-defined trade-offs between exploration and exploitation of the parameter space. This iterative non-parametric Bayesian approach is better able to handle uncertainty in parameter assignments than our previously used optimization techniques, which were based on local surrogate functions. The GP formalism also allows for predictions at test points using global information about the smoothness and self-similarity of the objective.

We simultaneously seek to expand the repertoire of data sources to which these methods can be applied, with specific focus on moving from the static light scattering (SLS) of prior work to small angle X-ray/neutron scattering (SAXS/SANS). SAXS has already proven valuable for reconstructing kinetics of capsid assembly systems (e.g. SV40 VP1 pentamers encapsidating short RNA molecules [23], and distinguishing closed shells from incomplete intermediates during P22 assembly [24]) while SANS has been applied to similar reconstruction problems of other protein assemblies, such as the Huntington amyloid [25]. Time-resolved SAXS has also been used to study the dynamics of conformational change in viruses [26–28].

Here, we develop and implement our GP optimization framework and demonstrate it using synthetic SAXS data of known ground truth. We implement both stochastic (SSA [29]) and deterministic (ordinary differential equation (ODE) [3]) models of virus-like assembly systems of known parameters. We then demonstrate that we can accurately reconstruct the original models from simulated SAXS data derived from these systems. While our stochastic and deterministic models are applicable to virus like assembly systems, the parameter inference framework is quite general and can be expected to be appropriate to any system for which model predictions are costly to evaluate and noisy.

## Methods

### Overview and objective

The overall goal of our method is to learn a set of model parameters, specifically kinetic rate constants for distinct self-assembly reaction events, that define a quantitative model of assembly that is maximally consistent with a set of experimental data. We assume the data here to be SAXS or SANS waveform data, explained below, which we will canonically reference as SAXS data. We particularly develop a class of methods designed to learn a stochastic process, which is utilized to obtain an optimal assembly model specification. The process probabilistically models an objective function quantifying the difference between a ground truth SAXS experiment, for which we have (synthetic) data, and a candidate experiment determined from a simulation trajectory at a single hypothetical point in parameter space.

We define our objective function as the root mean square deviation (RMSD) between the respective sets of intensity curves over designated time points. We generalize this objective for use with two model types in common use in this field, SSA-based stochastic models and ODE continuum models, each of which can work with the same basic inference framework with some specialized modifications. For the stochastic assembly framework, no two trajectories’ reactions will occur at the same time points. We can get around this problem because assembly is a Markov process in which the system state remains unchanged between any two successive reactions. Thus, for each time point in the ground truth experiment, we select the closest later time point from the candidate experiment when computing the RMSD. Within the continuous time ODE framework, we can directly specify the time points to consider via interpolation relative to a finite difference numerical integration. We note that we do not generalize to coarse-grained Brownian particle models despite their widespread use, because they cannot be parameterized straightforwardly in terms of reaction rate constants like SSA and ODE models.

Our central task in model inference is to approximately minimize this objective as efficiently as possible. Note that our use of stochastic processes to represent the objective means we are effectively specifying a probability density over possible rate parameters. This contrasts with our prior work [13, 14, 17, 19] fitting the rate parameters directly or with conventional Bayesian optimization that directly samples over the parameter space. While this may seem a complicated approach, this complication is the key to kriging methods gaining the advantages of a Bayesian model in estimating model uncertainty while simultaneously getting the high efficiency needed by the application. Noise variance in the stochastic case is significant and no single assembly trajectory, or resulting SAXS experiment, can be taken as representative for a given model specification. We determine the representative by simulating multiple trajectories, translating each into a SAXS experiment, and then taking their element-wise mean. The objective’s empirical noise level is also approximated by computing the RMSD of each repeated simulation and calculating their variance directly.

### Data sources

Scattering experiments consist of a wave source (x-rays/neutrons for SAXS/SANS, respectively) directed towards a sample. After interacting with the sample medium, some fraction of the incident waves scatter away while the remainder are absorbed. The intensity of scattered radiation is measured at a detector as a function of the scattering vector, *q*. Small-angle scattering roughly corresponds to measured intensities at low *q* values, allowing the investigation of microscopic features with spatial resolution ranging from a few angstroms to a few microns. Mathematically, the scattered intensity *I(q)* is the Fourier transform of the electron density correlation function, therefore signal is observed only if the contrast in electron density is different from zero. However, scattering experiments do not provide localized information about the sizes, shapes and pairwise distances of the molecular constituents. Instead, the intensity is representative of the entire sample, providing a spatial and temporal average over the duration of the measurement (temporal resolution as low as 100ps [30]). Due to these limitations, scattering provides only bulk, indirect evidence of assembly dynamics. See [30, 31] for a detailed treatment. There are also numerous examples of small-angle scattering with other protein systems, e.g. [32–38].

*In silico* SAXS experiments are constructed from simulated assembly trajectories by extrapolating the solution scattering of a single protein subunit obtained from CRYSOL. CRYSOL [39] is a program for evaluating the solution scattering from macromolecules with known atomic structure and accepts as input PDB structure files. The present work focuses on fixed structures for the dimer subunits of cowpea chlorotic mottle virus (CCMV) formed from the *A*−*B* and *C*−*C* chains (PDB 1za7, [40]), as well as a model of the pentamer subunits of generic dodecamer assembly. Clement et al. [41] examines the energetic effects of allowing the subunits to come from a distribution of possible configurations rather than a single PDB structure.

CRYSOL ouputs a vector of scattering intensities corresponding to *q* values from 0 to 0.5 in steps 0.01. As the higher *q* values correspond to observations of smaller features of the system, possibly beyond experimental reliability, there is a question as to the correct range to consider. However, in the context of our purely computational experiments we will not consider the issue and use the default range returned by CRYSOL.

The full SAXS intensity is determined as a function of the form factor, *F*(*q*), and the structure factor, *S*(*q*). Loosely speaking, the form factor is determined by the internal structure of the elementary particles in the system (i.e. the protein subunits), and the structure factor provides information on larger scale spatial correlations among the elementary particles.

1 $$  I^{SAXS}(q) = \Delta\rho^{2}V^{2}|F(q)|^{2}S(q)  $$

In Eq. 1, *V* is the elementary particle volume, and *Δρ* the electron density contrast between particle and solution. These two terms, together with the form factor, are returned by CRYSOL as the single subunit scattering. Because our simulators do not directly model diffusion through space, we do not have any information on the relative positions of the various intermediates present at each reaction step. Our mathematical extrapolation of the subunit scattering to the full system scattering therefore relies on a dilute assumption, allowing the scattering contributions of each intermediate to be summed. Within the context of a single intermediate, we do have access to relative subunit positions and so we calculate a structure factor for each.

2 $$  \begin{aligned} S(q) = \frac{1}{N} \Sigma_{j,k} e^{-iq(R_{j} - R_{k})} \end{aligned}  $$

In Eq. 2, *i* is the imaginary unit, $\sqrt {-1}$, and the summation is over every pair, (*j*,*k*), of the *N* subunits present in the intermediate, located at positions *R*_*j*_,*R*_*k*_. The full waveform, *I*^*SAXS*^(*q*), specified as a function of *q* over a defined range and step size, serves as the input to our model inference. One might in principle fit to multiple waveforms for a given system, for example from monitoring assembly at distinct concentrations, although for simplicity we assume here that we are fitting to a single SAXS experiment.

### Stochastic simulation model

The simulation-based data fitting approach used here depends on fitting a model to a data set through an intermediate simulation. That is, one assesses quality of fit of a parameter set based on how well the true experimental data matches simulated experimental data, derived by simulating assembly with the parameter set and then generating SAXS/SANS data from the output of that assembly simulation. In the present work, the “true” data is also simulated, which is necessary to have a data set with known ground truth. We implement two versions of the full pipeline here, one for a stochastic simulation class and one for a deterministic one, in each case using the same techniques for creating true data and for fitting to those data.

Stochastic simulations are run using DESSA [7] which implements a version of the Gillespie algorithm [29] for coarse grained, complex self-assembly systems [6]. Reaction chemistry is represented as a continuous time Markov model of the possible reaction trajectories (see [7] for details) available to an initial collection of protein subunits that undergo association and dissociation reactions according to a local rule model [4, 42]. The local rules describe interactions between protein subunits in terms of the positions, affinities, and kinetics of their binding sites, with the binding rate constants the only free parameters. This simulator does not explicitly model diffusion in space, nor is it based on a lattice or compartment model. Instead, the intra-capsid geometry is modeled through the local rules, with diffusion implicitly a function of the kinetic rates under the assumption that the system is well mixed.

Because DESSA deals directly with expected wait time constants for reactions (*T*) rather than reaction rate constants (*k*), conversion between the two is useful. The unimolecular case (e.g. dissociation of species *S*_1_:*S*_1_→2*S*_1_) is easy. The reaction rate *k*_*uni*_ has units $\frac {1}{[s]}$, so the expected wait time *T*_*uni*_ is simply the inverse of the rate constant. The bimolecular (e.g., association: *S*_1_+*S*_2_→*S*_1_:*S*_2_) molar reaction rate constant is defined as $k_{molar} = \frac {N_{A}*\Omega }{T_{bi}}$, where *Ω* is the system volume, *N*_*A*_ is Avogadro’s number and *T*_*bi*_ is the expected reaction waiting time, again allowing one to derive the rate constant from the inverse of the waiting time and vice versa. Finally, because the search space can span multiple orders of magnitude along each dimension, we work in log-scaled units. We specifically treat the ground truth (GT) parameter values *T*_*GT*_ as the origin of CCMV’s 10-dimensional parameter space, with the conversion between real space (T) and log-scaled space (x) given by $\textbf {T} = 10^{(log(\textbf {T}_{GT})/log(10) + \textbf {x})}$. Search points are constructed by the algorithm as modifications of the ground truth point which is located at **x**=(0,0,0,0,0,0,0,0,0,0). In all results figures, the **x** values of points are displayed, rather than their **T** values.

### ODE model

Simulating assembly using ODEs requires a distinct differential equation describing the time evolution of the concentration of each potential intermediate, from monomeric subunit to complete capsid. We began, like Endres & Zlotnick [43] and Misra & Schwartz [12], by considering a model of *T*=1 assembly from pentameric capsid subunits, for which only monomer/monomer and monomer/oligomer reactions are possible. We justify this restriction of the pathway space based on the observation that, except in cases of extremely high rates or concentrations [44], the equilibrium concentration of monomers is much larger than that of the intermediates. With this simplification, we were able to compute each species in the assembly tree (all of the structurally unique partial assemblies of a given size), their forward/backward reaction degeneracies, and their relative stabilities. Following [12], we represent the molar concentration of the *k*^*th*^ unique species of size *j* as [j,k], and represent the forward reaction degeneracy between (j,k) and (m,n) after monomer addition as $a^{m,n}_{j,k}$. The corresponding backward reaction degeneracy is $b^{j,k}_{m,n}$. The relative stability of (j,k) w.r.t. (m,n) is approximated as

3 $$ s^{m,n}_{j,k} = exp(-\Delta G*(c_{m,n} - c_{j,k})/RT)  $$

were *c*_*j*,*k*_ is the number of bonds formed within species (j,k).

The differential equation for the time evolution of [*j*,*k*] is as follows.

4 $$  {}{\begin{aligned} \frac{d[j,k]}{dt}& = k_{on} \Sigma_{m,n} \left(b^{j,k}_{m,n} s^{m,n}_{j,k} [m,n] - a^{m,n}_{j,k} O(m-j)[j,k][m-j]\right) \\ & \quad- k_{on} \Sigma_{p,q} \left(b^{p,q}_{j,k} s^{p,q}_{j,k} [j,k] - a^{j,k}_{p,q} O(j-p)[j-p][p,q]\right) \end{aligned}}  $$

In Eq. 4, *O*(*m*−*j*) denotes the symmetry of the monomer subunits (or oligomer subunits if *m*−*j*>=1 were allowed). The full set of these equations for all defined *j* and *k* define an ODE model for time evolution of the complete reaction system.

**Fig. 1:**
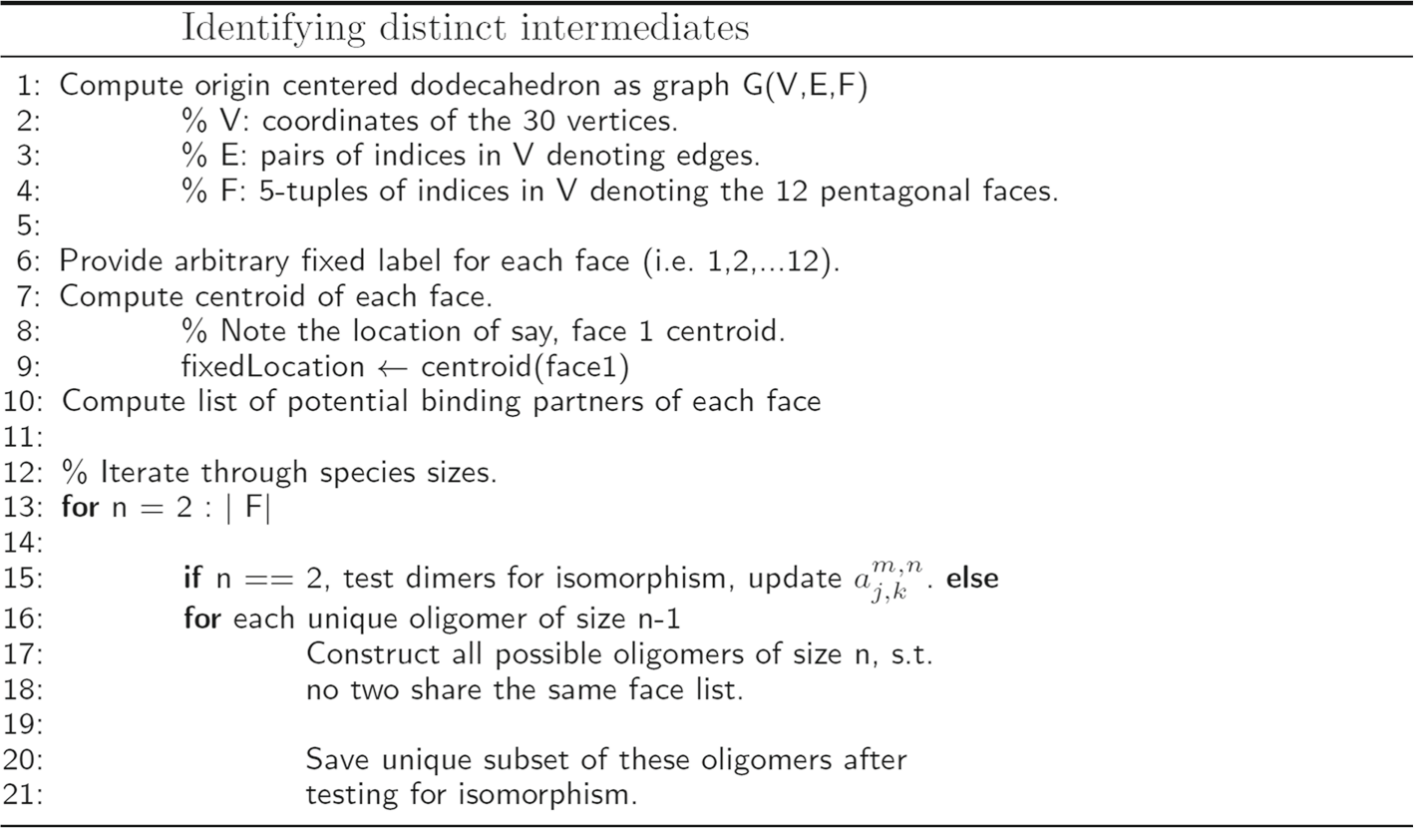
Pseudocode for identifying distinct intermediates

**Fig. 2:**
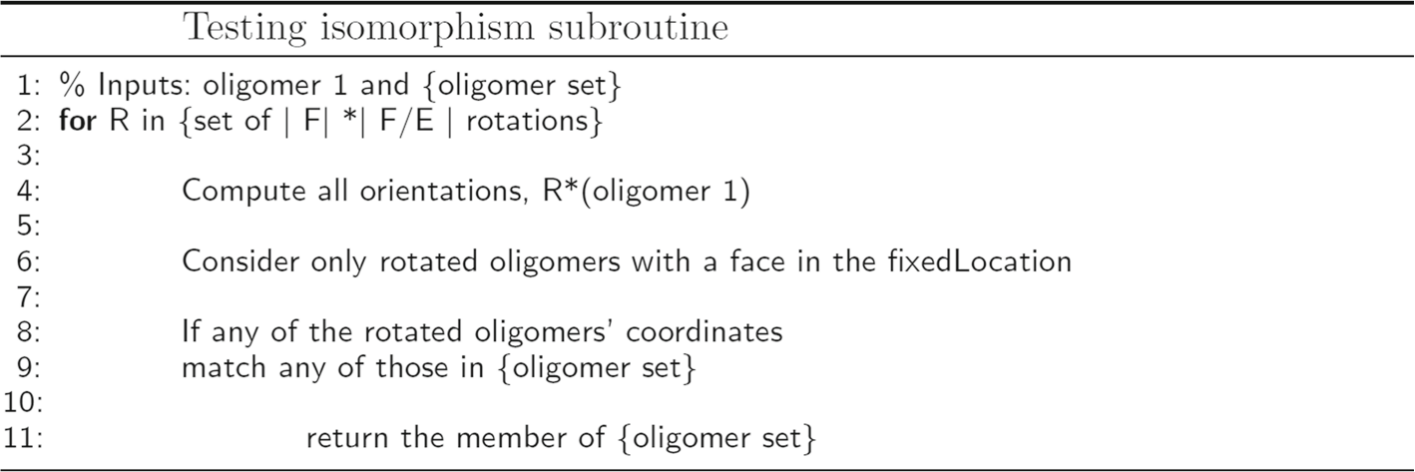
Pseudocode for determining if oligomers are isomorphic

For the equations to be correct, it is necessary to identify assemblies that are isomorphic to one another, a special case of the graph isomorphism problem. While a general algorithm for detecting isomorphism of subsets of icosahedral assemblies is provided in [12], we provide here an efficient variant customized for this application. Our new algorithm for identifying all structurally unique intermediate oligomers and computing the forward/backwards degeneracies for each relevant pair is shown in Fig. 1. It iteratively constructs the state space by adding a pentagonal monomer to each free binding site of the current oligomer, and tests the resulting oligomer set for isomorphism. Only the unique structures are saved, i.e. those which are not pairwise isomorphic under some transformation in SO(3). For each isomorphic structure generated, the appropriate $a^{m,n}_{j,k}$ is incremented. The isomorphism testing subroutine is outlined in Fig. 2. It relies on the fact that without loss of generality, we can enforce that all species contain the same initial monomer (which we call ’face 1’), and that the implicit dodecahedron – of which all oligomers are a part – can be oriented relative to a fixed location in space. For convenience, the centroid of face 1 is treated as the fixed location. There are 11 3D rotations leaving the dodecahedron in an orientation equivalent to the original, but which successively place each face in the fixed location. Further, for each of these orientations, due to the pentagonal symmetry of the subunits, there are 4 2D rotations leaving the centroid of the face in the fixed location unchanged. When determining if two oligomers are isomorphic, each of the 12*5 orientations of the first oligomer are computed successively and the resulting coordinates are compared with the second oligomer for identity. The isomorphism subroutine runs in time *O*(|*F*|∗|*E*/*F*|) where |*E*/*F*| is the number of edges per face. Finally, we note that many viruses possess icosahedral symmetry. Being dual to the dodecahedron and sharing the same symmetry group, the same set of rotations apply to the icosahedron.

### Gaussian process framework

#### Modeling the objective as a Gaussian process

Gaussian processes have a long history of use in disparate fields for related tasks including interpolation and prediction. For example, in geostatistics it has been known as kriging since the early 1970s [21]. The modern interpretation is that a GP assigns a probability distribution to a space of functions, the most important properties of which (e.g. smoothness) are determined by the GP covariance function. Due to its non–parametric nature, overfitting is less of a concern than it is with other regression models.

In analogy with the Gaussian distribution, the GP prior is completely defined by its mean function and covariance function [21].

5 $$ \begin{aligned} & F(x) \thicksim GP(m(x), k(x,x')) \end{aligned}  $$

The covariance function, *k*(*x*,*x*^′^), is of central importance. It defines a notion of similarity between points in the input space in terms of their objective values, enabling prediction at test points. It is technically a kernel function and must be symmetric. Further, when this kernel function is evaluated at a set of points, the resulting matrix must also be positive semidefinite. Combining the prior with new observations (i.e., training on pairs { **x**,*F*(**x**)}) leads to the posterior distribution over functions, representing our updated beliefs about possible candidate functions.

Once the form of the mean and covariance functions are specified initially, training is synonymous with covariance *hyper*parameter optimization (note that the model is still nonparametric because the hyperparameters define a class of GPs rather than a particular instance). In other words, the hyperparameters obtained are those that minimize the negative log marginal likelihood of the data under the GP class specified by the covariance function. This optimization step is usually very efficient and should not be confused with the larger optimization problem of minimizing the objective function.

### Gaussian process optimization

At a high level, the method will work iteratively by using the GP to identify candidate parameter sets at which to run additional simulations, which in turn are used to refine the GP fit. After training, the GP can be queried for the *best* new expensive point(s) to evaluate. This model includes uncertainty at test points, as it not only provides an estimate of the mean value of the objective, but also provides an estimate of the variance. Assuming the input space has not yet been thoroughly explored, the algorithm will benefit from objective evaluations at additional parameter points. In identifying candidate points to simulate, we need to balance exploration of unexplored (high variance) areas with exploitation of regions known to have low objective values. Several *acquisition functions* (AF) have been designed to handle this tradeoff at the expense of yet another (usually inexpensive relative to *F*(**x**)) optimization. We have chosen the lower confidence bound (LCB) as the acquisition function to be minimized due to its simplicity of evaluation.

6 $$  a_{LCB}(\textbf{x}) = \mu(\textbf{x}) - \kappa\sigma(\textbf{x})  $$

7 $$ \textbf{x}_{new} = {argmin}_{x}\; a_{LCB}(\textbf{x})  $$

In Eq. 6, *μ* denotes the mean prediction at each input and *σ* the corresponding standard deviation. The user-defined parameter *κ* balances the tradeoff (higher and lower for exploration and exploitation, respectively). It has been shown that choice of statistical model is often far more important than choice of acquisition function [45]. Other popular choices include *probability of improvement (PI)*, *expected improvement (EI)*, *entropy search*, and *Thompson sampling* [45, 46].

AF minimization can be achieved using derivative free optimization packages such as DIRECT [47, 48], SNOBFIT [49] and MCS [50], or methods such as sequential quadratic programming and quasi-Newton solvers. We, however, chose a simpler approach. We draw samples in relevant areas of the search space, evaluate the AF, and directly select the minimizer. This randomized procedure is repeated many times with the resulting set of minimizers coordinate-wise averaged.

#### Multi-GP model optimization

In many regression contexts, domain-specific knowledge can be applied to constrain the class of statistical model used to fit the experimental data. For example, it may be known from physical principles that observations should be distributed linearly with some corruption from random measurement error, suggesting the use of a linear regression model. In our case, GP regression allows a great deal more flexibility in principle but we lack prior knowledge about which, if any, GP class accurately models the process generating a particular set of observations. With sufficient training data, the kernel maximizing the likelihood of those data while also predicting the correct noise level is often the best choice. However, our focus is optimization of the objective with as few function evaluations as possible. A novelty of the proposed method is the assumption that using multiple statistical models for experimental data generation at once, rather than in a one-off fashion, may allow us to more efficiently discover structure, e.g., the locations of local minima. Additionally, during early rounds of search, this strategy can provide an avenue for more thoroughly exploring the input space since the acquisition functions corresponding to different kernels may be minimized by different points.

The kernel functions used in the present work are listed in Table 1. We sought to include a range of traditional kernels as well as a few less common choices. All except the Neural Network (NN) covariance are stationary in the sense of depending on the relative difference **x**−**x’** rather than on the absolute locations in parameter space. The Square Exponential (SE) covariance leads to extremely smooth candidate functions (i.e., infinitely differentiable). This smoothness may not be realistic for objective functions associated with physical processes, but it is the most widely used in machine learning. The Matern class covariance with hyperparameter *ν*=7/2 (not used) leads to candidate functions very similar to the SE. As *ν* moves through 5/2 and 3/2, the respective candidate functions become rougher. Values of *ν* below 3/2 are not recommended for regression, and non half-integer values lead to very complicated forms for *k*(*x*,*x*^′^). The Rational Quadratic (RD) kernel can be viewed as a mixture of many SE kernels, each with a distinct lengthscale hyperparameter, and is very general. The NN covariance allows us to perform regression with the equivalent of an infinitely wide single layer network using the error function as the hidden unit. The Gabor covariance enables the discovery patterns in the data which incorporate some periodicity, and extrapolation based on the pattern. Our decision to include these kernels and not others is somewhat arbitrary beyond the fact that they impose a diversity of assumptions on data generation. Future work may consider more principled methods for the number and types of kernel to be used.

**Table 1:** Kernel functions, *k*(*x*_1_,*x*_2_)

1. Matern 3/2 (ARD): $\sigma ^{2}(1+\sqrt {3}\sqrt {r}) * exp[-\sqrt {3}\sqrt {r}]$
2. Matern 5/2 (ARD): $\sigma ^{2}(1+\sqrt {5}\sqrt {r}+(5r)/3) * exp[-\sqrt {5}\sqrt {r}]$
3. Rational Quadratic (ARD): *σ*^2^(1+*r*/(2*α*)^−*α*^
4. Rational Quadratic (ISO): *σ*^2^(1+*s*/(2*α*)^−*α*^
5. Gabor (ARD): $h(x_{1} \!\,-\, x_{2}); h(t) \,=\, exp[\,-\,\sum \!((t.^{2})./(diag(P.^{2})]*cos[\!2\pi \!\sum \!(t./p)]$
6. Neural Network: $\sigma ^{2} \arcsin {\left [x_{1}^{T}P x_{2} / \sqrt {\left (1 + x_{1}^{T}P x_{2}\right)*\left (1 + x_{1}^{T}P x_{2} \right) }\right ]} $
7. Square Exponential (ARD): *σ*^2^*exp*[−*r*/2]
*r*=(*x*_1_−*x*_2_)^*T*^∗*P*^−1^∗(*x*_1_−*x*_2_);*s*=(*x*_1_−*x*_2_)^*T*^∗(*ℓ*∗*I*)^−1^∗(*x*_1_−*x*_2_)
*P* is the diagonal matrix of ARD lengthscale hyperparameters.
*ℓ* is a scalar lengthscale hyperparameter; *I* is the unit matrix.
*α* is a shape hyperparameter for the rational quadratic kernel.
*p* is a vector of period hyperparameters.

**Fig. 3:**
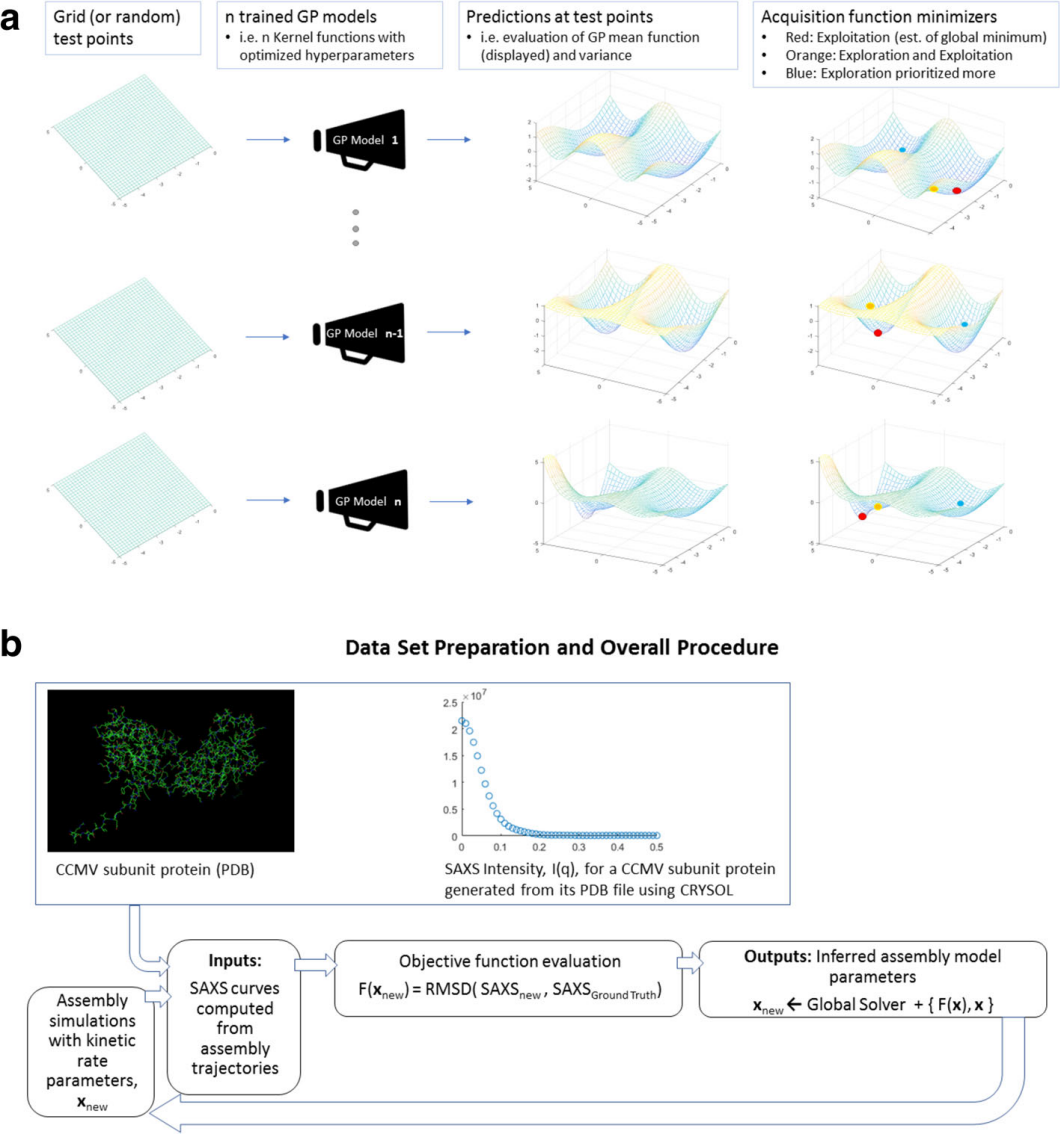
High-level overview of our multi-GP optimization strategy. **a** Visualization of a single round of our multi-GP model optimization. **b** Overall parameter inference pipeline incorporating the multi-GP optimization method of **a**

Figure 3a visualizes the main aspects of our global optimization method during a single round of search and (b) illustrates how the optimization fits into the overall data set preparation and parameter inference pipeline.

## Results

Gaussian process model specification and hyper-parameter optimization was performed using code released by Rasmussen, Nickisch, Williams and Duvenaud [51, 52].

### Stochastic simulation model results

We begin the model fitting by sampling a selection of points in parameter space uniformly at random from a hypersphere, a contrast to our earlier methods that begin with a regular grid search [14] that is motivated by prior work showing random sampling to be more efficient when the objective surface has low effective dimensionality compared to the parameter space [53]. For the present experiments, the hypersphere is centered on the ground truth point. For each sampled point, we run a set of simulation trajectories, project SAXS outputs, and compute the associated RMSDs relative to the input data. The resulting data points are then used in initial GP kernel hyperparameter training, updating the prior over objective functions to a posterior. In subsequent rounds of search, the posterior density estimated by the GP from the previous round becomes the new prior density from which we select further parameter points for evaluation to produce an updated posterior.

To provide a comparison with a more traditional solver, we used SNOBFIT (Stable Noisy Optimization by Branch and FIT) [49] a Matlab-based solver that combines a branching strategy with localized quadratic response surface fitting for fast, continuous optimization of black box functions satisfying a number of technical and design criteria. We favor SNOBFIT based on prior work showing it to be effective on capsid assembly simulation [17]. Its major advantage over competitor methods, including early stochastic process based methods such as DACE and SPACE [54, 55] as well as more traditional iterative modeling methods such as DIRECT and UOBYQA [56, 57], is its ability to handle all of the following cases: function values are expensive to evaluate; function values may be available only at approximately the requested points; the function values are noisy; the objective is non-convex; no gradients are available; there are hidden constraints; there are soft constraints; parallel function evaluation is desired; function values may be obtained extremely infrequently; and, the objective function or the search region may change during optimization. In the present work, the comparison is in terms of the number of function evaluations necessary to recover the ground truth parameter vector. Each time SNOBFIT is called, it uses function evaluations from previous rounds as well as newly evaluated points to return a user-specified number of function minimizers to be evaluated evaluated in the next round. These minimizers belong to one of 5 classes: 1-3 being local estimates, and 4-5 global estimates. Plots indicate the local/global classification of each returned point.

**Fig. 4:**
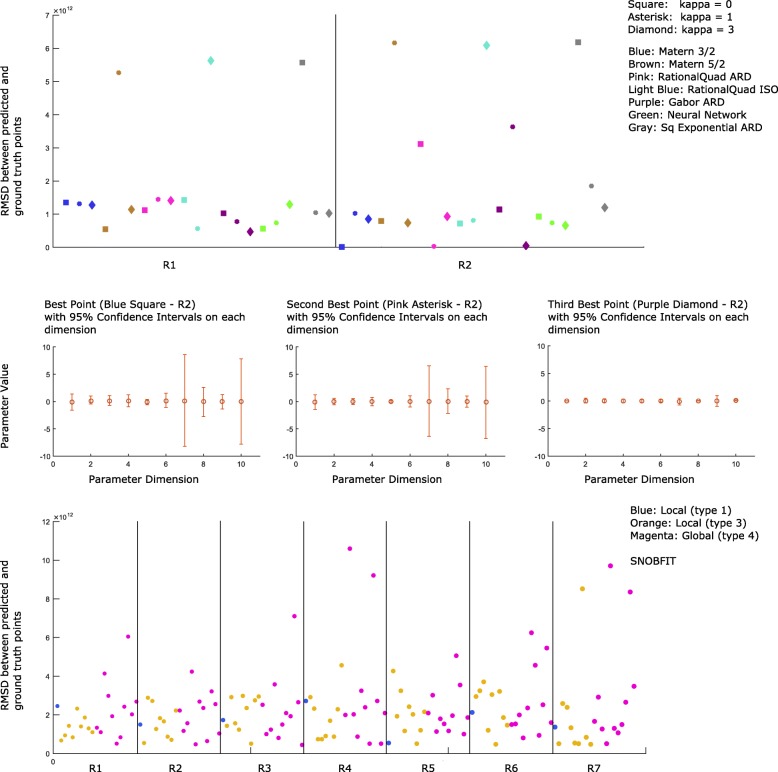
Comparison of objective values for our multi-GP optimization (top/middle) and SNOBFIT (bottom). Both methods use the same training set of 100 randomly sampled inputs, and both return 21 points for evaluation in a subsequent round. In the second round (R2 region) of search, our method recovers 3 low RMSD points, i.e. the blue square, pink asterisk and purple diamond. These three points, displayed in the middle figures with 95% confidence intervals for each dimension, minimize acquisition functions for distinct GPs and exploration/exploitation trade-off parameters. Displayed for SNOBFIT are 7 rounds of search in which it fails to recover equally low RMSD points. With default settings, SNOBFIT returned points of three types (distinguished by color), two of which result from local searches, and the remaining from a global search

We first show results of a search of a small parameter space, corresponding to a hypersphere of radius 3 logs around the ground truth. For this search, we used an initial sample of 100 points, with 300 trajectories per point sampled. Figure 4 shows RMSD as a measure of search progress for our method (top/middle) and for SNOBFIT (bottom). The *κ* parameter listed for our method balances the degree to which the search favors exploration of uncertain regions (higher *κ* values) versus exploitation of low variance regions. After one round of optimization, no kernel is clearly superior in discovering the correct parameter set. After two rounds, in each case one of the seven kernels shows a near optimal fit, although it is surprisingly a different kernel for each choice of *κ*. The three best scoring points are displayed in Fig. 4 (middle) with 95% confidence intervals on each dimension. These intervals are kernel-dependent and so we used the kernel responsible for recovering the point in their calculation. To estimate confidence intervals in a particular dimension, we use the GP model to sample a series of RMSD values from a sequence of points in the vicinity of the chosen optimum in that dimension, with the spacing for the sequence determined in part by the applicable kernel lengthscale hyperparameter, *l*. Specifically, we scanned a region of 20*l* at a density of 0.001*l*. The regression provided us with predictions of *μ*(*RMSD*) and *σ*^2^(*RMSD*) at each point in the sequence from which we drew 10,000 random samples to estimate the fraction of times each point in the sequence would be predicted to yield the optimum RMSD. We then chose the minimal symmetric window of points around the optimum so as to account for 95% of the probability density of minimum RMSDs, providing an estimated 95% confidence interval.

**Fig. 5:**
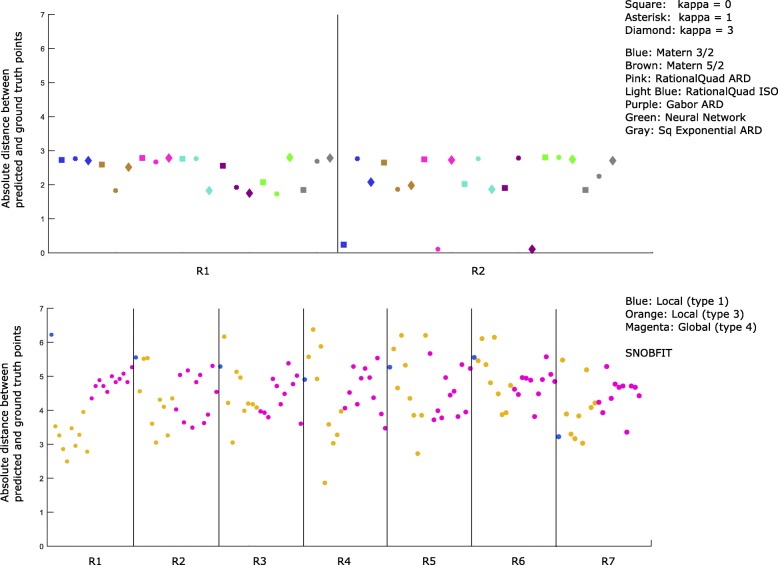
Comparison of error in predicted parameters for our multi-GP optimization (top) and SNOBFIT (bottom). Both methods use the same training set of 100 randomly sampled inputs, and both return 21 points for evaluation in a subsequent round. In the second round (R2 region) of search, our method recovers 3 points very close to the ground truth, i.e. the same blue square, pink asterisk and purple diamond. Displayed for SNOBFIT are 7 rounds of search in which it fails to recover equally close points

SNOBFIT with default settings was unable to recover low RMSD parameter sets from either its locally weighted (classes 1 and 3) or global (class 4) optimizations after seven rounds. Figure 5 shows another measure of search progress, the distance between predicted parameter points and the ground truth parameter set. Here we can see that the same points that approximately minimized the RMSD are also in fact close to the ground truth.

**Fig. 6:**
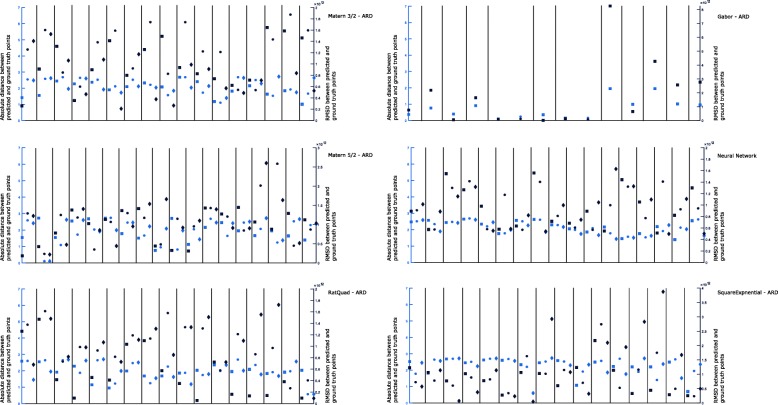
Results for single-GP parameter searches in which a single kernel function is used during all rounds. Displayed for each search are the errors in objective value (RMSD - right y-axis, dark blue) and error in predicted parameters (Distance - left y-axis, light blue) for each round

We further sought to compare the results to a more conventional kriging search by evaluating how well the method would perform using only a single kernel. Figure 6 are search results for which only a single kernel is used across all rounds of optimization, with the same 100-point training set as in Figs. 4 and 5. In the multi-GP search, it was the Matern 3/2 kernel that discovered the lowest RMSD (1.6×10^10^±2.2×10^11^) point after 121 total function evaluations. In the single-GP searches, the Gabor-ARD kernel was able to obtain the slightly lower value of 0.7×10^10^±2.2×10^11^ after only 106 total function evaluations. The remaining searches produced minima in the range 5.2×10^10^ – 2.4×10^11^ with similar noise levels. We can conclude that while a single kernel may lead to slightly better performance when seeking the minimum of a particular objective function, it is not obvious how to select this kernel beforehand, or to what extent the choice depends on the particular training examples seen. The multi-kernel approach does nearly as well as the best single-kernel approach without the need for advanced knowledge of how to select an appropriate kernel for a particular system.

**Fig. 7:**
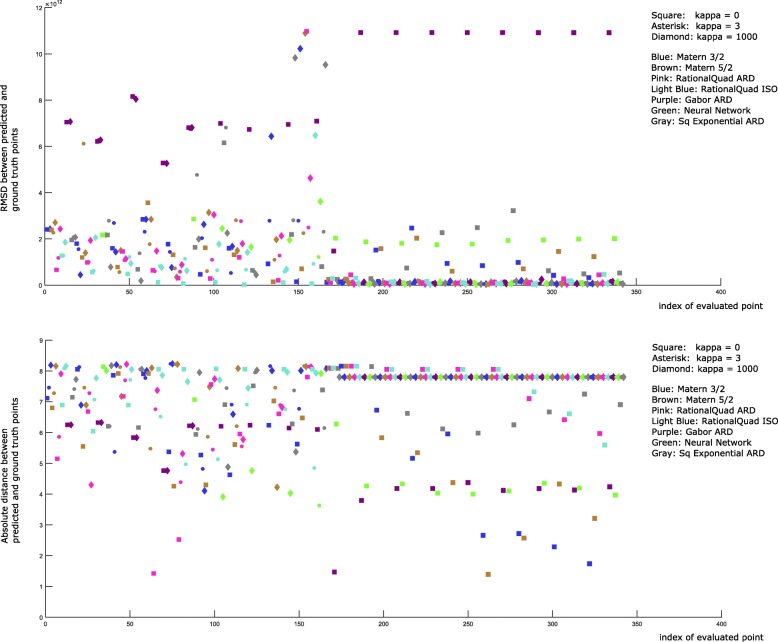
Results from 20 rounds of optimization for a large (hypersphere of radius 9 logs) search space. Individual rounds are not delineated due to variable numbers of points returned from acquisition function minimization in different rounds. Shown are (top) the RMSD values, and (bottom) the errors in predicted parameter sets as the search progresses. Note that the best points found (very low RMSD) often correspond to comparatively distant parameter sets. This is a result of the fact that the objective can be insensitive to large displacements in some of the input dimensions but not others

We next consider a search of a larger space, corresponding to a hypersphere of radius 9 logs using 71 initial training points and 100 trajectories per point sampled. For this search, we allowed the optimization to run for 20 rounds. The results show fairly high concordance among solutions, although with high variability in estimates of parameters p7 and p10. The results suggest the method is effective at finding low-RMSD solutions, although as might be expected, the solutions are sensitive only to a subset of the parameters. Figure 7 summarizes search progress to this point.

**Fig. 8:**
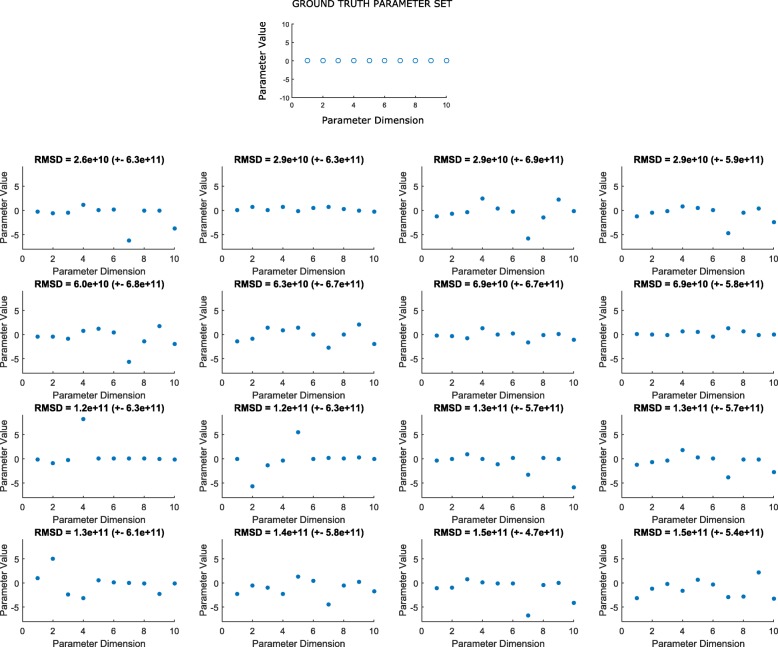
Sixteen points returned by re-evaluation selection subroutine. The points were chosen from among the 50 top scoring points over all rounds

**Fig. 9:**
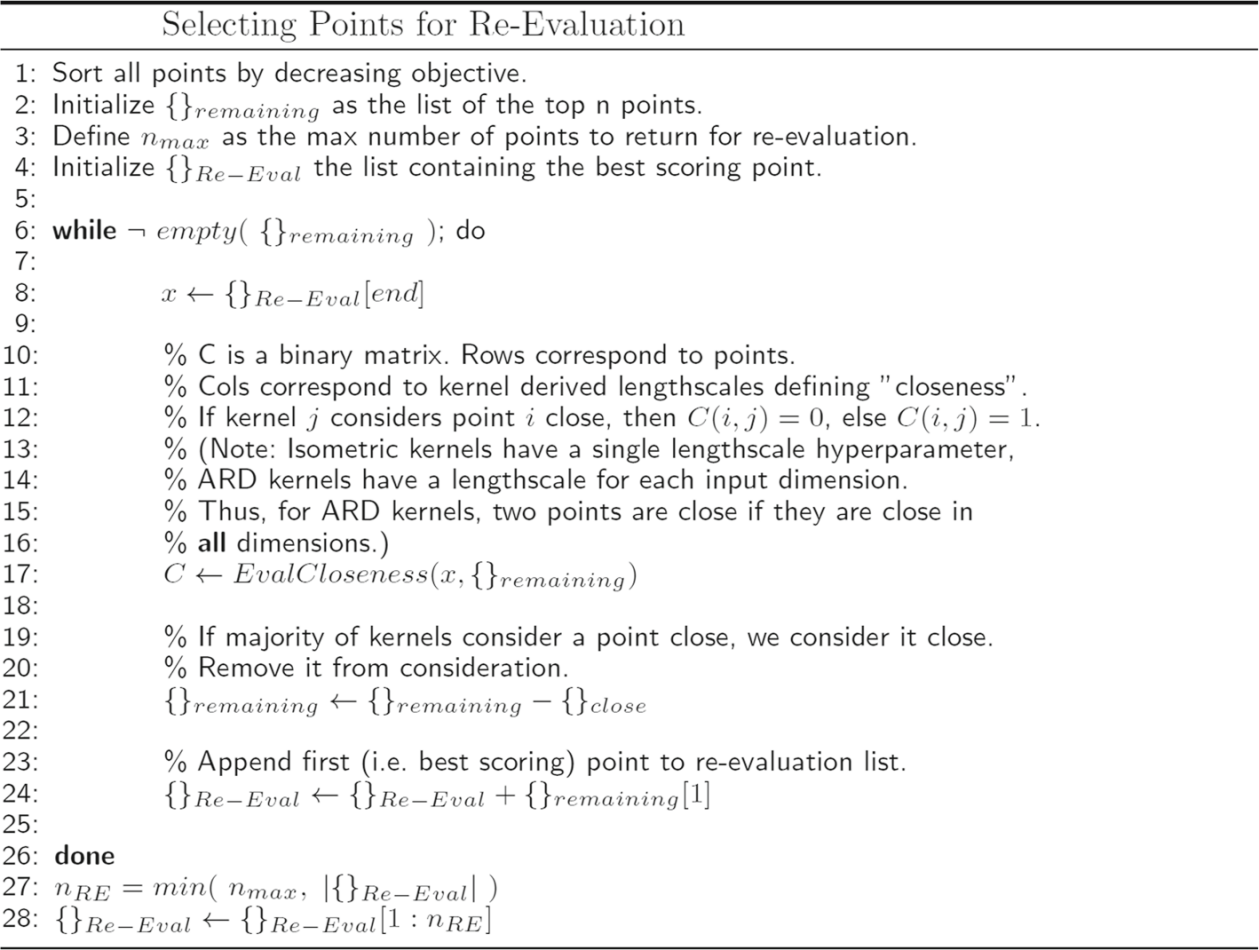
Pseudocode for selecting previously evaluated low RMSD points for re-evaluation at a lower noise level

As the predictions for each kernel begin to repeat round after round (e.g. beginning shortly after the 150th function evaluation in Fig. 7), it may be useful to re-evaluate points the algorithm deems good. As more simulated experiments are averaged at a point, the corresponding objective becomes more accurate, potentially allowing better discrimination between similarly good points and better generalization at nearby points. We have settled on a number of criteria for the selection of the most useful set of points to re-evaluate with more simulations. First, the set should be a subset of previously evaluated points. This allows the utilization of previously run simulations. Second, no two points should be “close” (as defined by a kernel’s lengthscale hyperparameter). Third, the set should prioritize higher scoring points. These criteria suggest a selection subroutine analogous to agglomerative, hierarchical clustering, with the highest scoring cluster representatives chosen for re-evaluation. The resulting set of points was limited to 16 and is shown in Fig. 8. See Fig. 9 for pseudocode of the selection subroutine. In this case, re-evaluation of each of the 16 points with 1000 additional simulated experiments did not alter their relative ordering. It is interesting to note that for the smaller search space, the lowest RMSD points tend to also be closest to the ground truth in Euclidean distance. However, for the larger search space, the lowest RMSD are among the furthest from ground truth. This is again an illustration of the fact that the objective function is not equally sensitive to changes in each dimension. To obtain more precise estimates of the global minimum, one strategy would be to begin new small (e.g. hyperspheres of radius 3 logs) searches at low noise levels, and centered successively on each of the top 16 points.

### ODE model results

We next examined the utility of the solver for deterministic optimization using an ODE model of capsid assembly represented as a dodecamer, as in [43]. Here, we follow the assumption that each step in the oligomerization reaction may have an independent rate, but equating all oligomers of a given size. That is, we assume there is a single oligomer of size *N* that has a defined rate of transition to size *N*+1, but allow that the transition from *N* to *N*+1 may have a different rate than that from *N*^′^ to *N*^′^+1 for *N*≠*N*^′^. We here examine two cases: a 6 parameter model (grouping [1,2],...[11,12]) and the full 12 parameter model in which each oligomer has its own on-rate. We arbitrarily define the ground truth for the differential equation model to be a parameter vector in which each element (reaction rate) has the value 100 (in real space as opposed to the log space used in the stochastic simulations) and we conduct the parameter search in a hypersphere of radius 100 around this ground truth value.

**Fig. 10:**
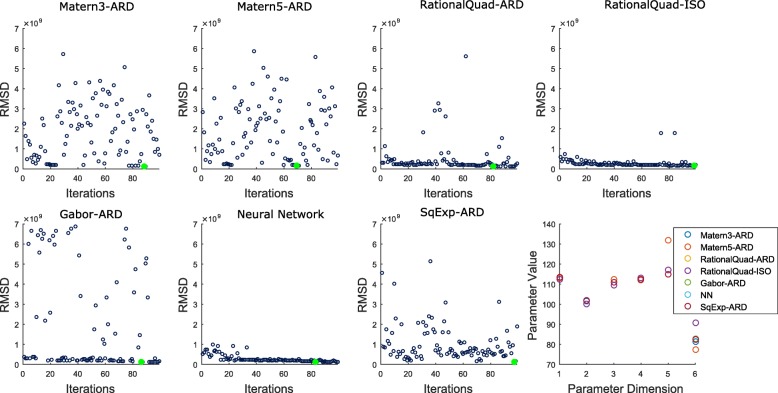
Model-fitting results for a 6-parameter ODE model. The results reflect 100 rounds of search with 893 points evaluated following initial training on 200 points randomly selected from a radius 100 hypersphere. Each round results in a new predicted minimizer for each GP model. The correspoinding RMSD is displayed. The lowest RMSD for each GP model over all rounds is plotted as a large green filled circle. In the final subfigure, the inputs with the lowest RMSD for each GP model are displayed

**Fig. 11:**
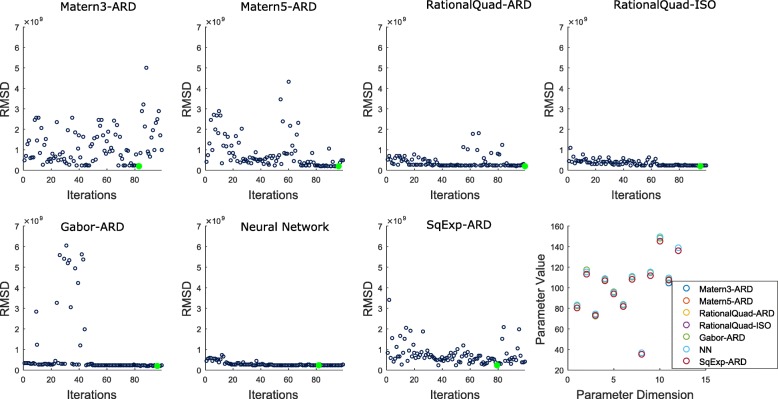
Model-fitting results for a full 12-parameter ODE model. The results reflect 100 rounds of search (893 points evaluated following initial training on 200 points randomly selected from a radius 100 hypersphere). Each round results in a new predicted minimizer for each GP model. The corresponding RMSD is displayed. The lowest RMSD for each GP model over all rounds is plotted as a large green filled circle. In the final subfigure, the inputs with the lowest RMSD for each GP model are displayed

Figures 10 and 11 show the results of the six and twelve parameter models. Each subfigure shows RMSD as a function of the number of search rounds for each kernel. We note that the ground truth in each case has an RMSD of exactly zero, yet moving a small distance away necessitates a minimal RMSD in the realm of 10^7^ due to the way SAXS experiments are evaluated. The objective surface is roughly constant in a neighborhood surrounding ground truth, with a very steep descent in its immediate vicinity. Thus, we should expect the accuracy of the approximate global minimizer to depend on the size of this surrounding neighborhood. In each assembly model (6 or 12 parameters), different kernel functions are able to identify this neighborhood with varying amounts of training data.

**Fig. 12:**
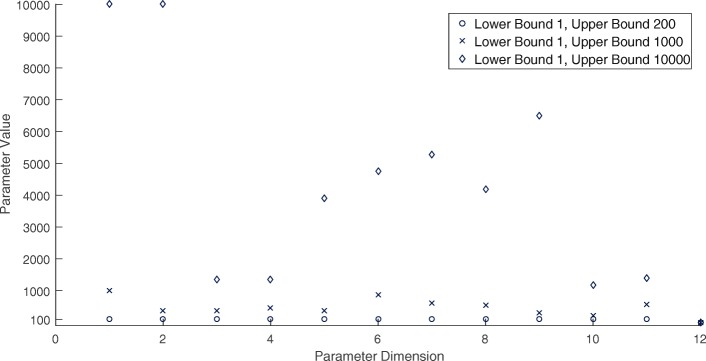
Results for ODE model-fitting using three separate MCS searches with varying search space sizes: 200 (1200 function calls, max allowed 7200), 1000 (878 function calls, max allowed 7200), and 10,000 (1439 function calls, max allowed 7200). Each dimension is lower-bounded by 1 to enforce that all rate constants are positive. Only the smallest search space resulted in the correct minimizer being found

To provide comparison to a competitive existing black box global minimizer, we use Multilevel Coordinate Search (MCS) [50], a more appropriate choice than SNOBFIT when solving for a deterministic objective. MCS is based on the DIRECT method and can be classified as *branch without bound* in the sense that it sequentially partitions the search space. As an improvement on DIRECT, the balance between global and local search is handled through a multilevel approach (partitioning the space along a single coordinate only). The method is guaranteed to converge if the objective is continuous in the neighborhood of a global minimizer. Because MCS is designed as a MATLAB caller, taking the black box function as an input, we were not able to easily asses its performance in terms of the number of function evaluations. Rather, it runs until convergence (or a stopping criteria is met) and outputs the minimizer, objective value, number of function evaluations, number of function evaluations used in local search, and other algorithm parameters. Figure 12 shows the results of MCS searches of increasing search space size. The ground truth is again a 12D vector with each element 100. The plot shows that MCS performs well when we have relatively tight bounds on the global minimum, in fact far better than our GP method, but poorly when those bounds are relaxed. A good strategy for solving deterministic systems of similar dimension may therefore be to narrow down the search region using the GP approach, and then apply MCS for a more accurate solution.

## Discussion

While our work provides a proof-of-concept demonstration of the multi-GP strategy, it offers many avenues for improvement. For example, our current goal is efficient global optimization with respect to the number of function evaluations, yet when considering the vast variation in resources required for a given evaluation (in terms of simulation time as well as memory), it may make sense to define efficiency with respect to total search time instead. To give some idea of the time required for stochastic assembly, evaluation of the ground truth point with 300 trajectories takes on the order of 30 min, while distant points in parameter space can span the range of hours to a week. One way to accomplish this may be to separately model the expected evaluation time, and take this into account during AF minimization. Another avenue for improvement concerns the empirical noise variance in RMSD at evaluated points; information to which we have access but do not directly utilize in GP regression. Modeling this variance itself as a GP may improve the ability of the LCB, which is constructed with the standard deviation at test points, to explore the space.

Furthermore, like all black box search methodologies, ours requires many design choices which balance competing factors including run time, cluster architecture, available memory, and the details of simulating molecular assembly. We attempted to bias the search as little as possible, defining the search bounds as a hypersphere surrounding the known ground truth and selecting initial training points randomly within the region, and refraining from enforcing hyper-priors on the kernel hyperparameters. In acquisition function minimization our sampling methods were simple, again based around randomly selecting points from hyperspheres, and more sophisticated sampling strategies might lead to more efficient optimization.

Finally, it also important to note that this method is limited to learning models of a system under experimental conditions, typically in vitro, which may be quite far from conditions of the functional system in vivo. Many extrinsic factors might perturb system behavior in vivo, such as the presence of other molecules interacting with the system or generic effects, such as molecular crowding. Prior work has explored the question of how to “correct” a rule-based system learned in vitro for some effects one would expect in vivo (e.g., crowding [19]). Such approaches cannot account for all possible differences, though, and addressing that issue is a hard problem that would need to be solved on a system-specific basis.

## Conclusions

We develop a novel method for efficient Bayesian parameter inference from rule-based models of molecular self-assembly and demonstrated it for fitting stochastic and deterministic models of viral self-assembly to simulated SAXS or SANS data. Our results show that for stochastic systems of low to moderate dimension, treating the objective function as being separately generated by multiple Gaussian processes can be an effective way to discover its structure. When placed within a Bayesian optimization framework, this translates to efficiently discovering locally optimal regions of the parameter space.

## Abbreviations

AF: acquisition function
CCMV: cowpea chlorotic mottle virus
DFO: derivative-free optimization
EI: expected improvement
GP: Gaussian process
LCB: lower confidence bound
MCS: Multilevel coordinate search
ODE: ordinary differential equation
PDB: protein data bank
PI: probability of improvement
RMSD: root mean square deviation
SAXS/SANS: small-angle x-ray/neutron scattering
SLS: static light scattering
SNOBFIT: Stable noisy optimization by Branch and FIT
SSA: stochastic simulation algorithm

## References

[1] Karr JR, Sanghvi JC, Macklin DN, Gutschow MV, Jacobs JM, Bolival B, Assad-Garcia N, Glass JI, Covert MW (2012). A whole-cell computational model predicts phenotype from genotype. Cell.

[2] Le Novère N (2015). Quantitative and logic modelling of molecular and gene networks. Nat Rev Genet.

[3] Zlotnick A (1994). To build a virus capsid: an equilibrium model of the self assembly of polyhedral protein complexes. J Mol Biol.

[4] Schwartz R, Shor PW, Prevelige PE, Berger B (1998). Local rules simulation of the kinetics of virus capsid self-assembly. Biophys J.

[5] Rapaport D, Johnson J, Skolnick J (1999). Supramolecular self-assembly: molecular dynamics modeling of polyhedral shell formation. Comput Phys Commun.

[6] Jamalyaria F, Rohlfs R, Schwartz R (2005). Queue-based method for efficient simulation of biological self-assembly systems. J Comput Phys.

[7] Zhang T, Rohlfs R, Schwartz R. Implementation of a discrete event simulator for biological self-assembly systems. In: ME Kuhl, NM Steiger, FB Armstrong, JA Joines, editors. Proc. Winter Simulation Conference; Orlando, FL. 2005;:2223–31. Winter Simulation Conference.

[8] Hagan M. F, Chandler D (2006). Dynamic pathways for viral capsid assembly. Biophys J.

[9] Hemberg M, Yaliraki S. N, Barahona M (2006). Stochastic kinetics of viral capsid assembly based on detailed protein structures. Biophys J.

[10] Keef T, Micheletti C, Twarock R (2006). Master equation approach to the assembly of viral capsids. J Theor Biol.

[11] Nguyen HD, Reddy VS, Brooks CL (2007). Deciphering the kinetic mechanism of spontaneous self-assembly of icosahedral capsids. Nano Lett.

[12] Misra N, Lees D, Zhang T, Schwartz R (2008). Pathway complexity of model virus capsid assembly systems. Comput Math Methods Med.

[13] Kumar MS, Schwartz R (2010). A parameter estimation technique for stochastic self-assembly systems and its application to human papillomavirus self-assembly. Phys Biol.

[14] Xie L, Smith GR, Schwartz R (2017). Derivative-free optimization of rate parameters of capsid assembly models from bulk in vitro data. IEEE/ACM Trans Comput Biol Bioinforma.

[15] Thomas M, Schwartz R (2017). Quantitative computational models of molecular self-assembly in systems biology. Phys Biol.

[16] Wilkinson DJ (2007). Bayesian methods in bioinformatics and computational systems biology. Brief Bioinform.

[17] Xie L, Smith GR, Feng X, Schwartz R (2012). Surveying capsid assembly pathways through simulation-based data fitting. Biophys J.

[18] Conn AR, Scheinberg K, Vicente LN (2009). Introduction to Derivative-free Optimization.

[19] Smith GR, Xie L, Lee B, Schwartz R (2014). Applying molecular crowding models to simulations of virus capsid assembly in vitro. Biophys J.

[20] Smith GR, Xie L, Schwartz R (2016). Modeling effects of rna on capsid assembly pathways via coarse-grained stochastic simulation. PloS ONE.

[21] Rasmussen CE, Williams CK (2006). Gaussian Processes for Machine Learning.

[22] Kleijnen JP (2009). Kriging metamodeling in simulation: A review. Eur J Oper Res.

[23] Kler S, Asor R, Li C, Ginsburg A, Harries D, Oppenheim A, Zlotnick A, Raviv U (2012). Rna encapsidation by sv40-derived nanoparticles follows a rapid two-state mechanism. J Am Chem Soc.

[24] Tuma R, Tsuruta H, French KH, Prevelige PE (2008). Detection of intermediates and kinetic control during assembly of bacteriophage p22 procapsid. J Mol Biol.

[25] Stanley CB, Perevozchikova T, Berthelier V (2011). Structural formation of huntingtin exon 1 aggregates probed by small-angle neutron scattering. Biophys J.

[26] Canady MA, Tsuruta H, Johnson JE (2001). Analysis of rapid, large-scale protein quaternary structural changes: time-resolved x-ray solution scattering of nudaurelia capensis *ω* virus (n *ω*v) maturation. J Mol Biol.

[27] Lee KK, Tsuruta H, Hendrix RW, Duda RL, Johnson JE (2005). Cooperative reorganization of a 420 subunit virus capsid. J Mol Biol.

[28] Matsui T, Tsuruta H, Johnson JE (2010). Balanced electrostatic and structural forces guide the large conformational change associated with maturation of t = 4 virus. Biophys J.

[29] Gillespie DT (1977). Exact stochastic simulation of coupled chemical reactions. J Phys Chem.

[30] Chaudhuri BN (2015). Emerging applications of small angle solution scattering in structural biology. Protein Sci.

[31] Glatter O, Kratky O (1982). Small Angle X-ray Scattering.

[32] Vestergaard B, Groenning M, Roessle M, Kastrup JS, Van De Weert M, Flink JM, Frokjaer S, Gajhede M, Svergun DI (2007). A helical structural nucleus is the primary elongating unit of insulin amyloid fibrils. PLoS Biol.

[33] Sato D, Ohtomo H, Yamada Y, Hikima T, Kurobe A, Fujiwara K, Ikeguchi M (2016). Ferritin assembly revisited: a time-resolved small-angle x-ray scattering study. Biochemistry.

[34] Cammarata M, Levantino M, Schotte F, Anfinrud PA, Ewald F, Choi J, Cupane A, Wulff M, Ihee H (2008). Tracking the structural dynamics of proteins in solution using time-resolved wide-angle x-ray scattering. Nat Methods.

[35] Jensen MH, Toft KN, David G, Havelund S, Pérez J, Vestergaard B (2010). Time-resolved saxs measurements facilitated by online hplc buffer exchange. J Synchrotron Radiat.

[36] Graceffa R, Nobrega RP, Barrea RA, Kathuria SV, Chakravarthy S, Bilsel O, Irving TC (2013). Sub-millisecond time-resolved saxs using a continuous-flow mixer and x-ray microbeam. J Synchrotron Radiat.

[37] Kathuria SV, Guo L, Graceffa R, Barrea R, Nobrega RP, Matthews CR, Irving TC, Bilsel O (2011). Minireview: Structural insights into early folding events using continuous-flow time-resolved small-angle x-ray scattering. Biopolymers.

[38] Diaz JF, Andreu JM, Diakun G, Towns-Andrews E, Bordas J (1996). Structural intermediates in the assembly of taxoid-induced microtubules and gdp-tubulin double rings: time-resolved x-ray scattering. Biophys J.

[39] Svergun D, Barberato C, Koch MH (1995). Crysol–a program to evaluate x-ray solution scattering of biological macromolecules from atomic coordinates. J Appl Crystallogr.

[40] Speir JA, Bothner B, Qu C, Willits DA, Young MJ, Johnson JE (2006). Enhanced local symmetry interactions globally stabilize a mutant virus capsid that maintains infectivity and capsid dynamics. J Virol.

[41] Clement N, Rasheed M, Bajaj CL (2018). Viral capsid assembly: A quantified uncertainty approach. J Comput Biol.

[42] Berger B, Shor PW, Tucker-Kellogg L, King J (1994). Local rule-based theory of virus shell assembly. Proc Natl Acad Sci.

[43] Endres D, Miyahara M, Moisant P, Zlotnick A (2005). A reaction landscape identifies the intermediates critical for self-assembly of virus capsids and other polyhedral structures. Protein Sci.

[44] Zhang T, Schwartz R (2006). Simulation study of the contribution of oligomer/oligomer binding to capsid assembly kinetics. Biophys J.

[45] Shahriari B, Swersky K, Wang Z, Adams RP, de Freitas N (2016). Taking the human out of the loop: A review of bayesian optimization. Proc IEEE.

[46] Snoek J, Larochelle H, Adams RP (2012). Practical bayesian optimization of machine learning algorithms. F Pereira, CJC Burges, L Bouttou, KW Weinberger, editors. Proc. Advances in Neural Information Processing Systems.

[47] Jones DR (2001). Direct global optimization algorithm. Encyclopedia of Optimization.

[48] Brochu E, Cora VM, De Freitas N. A tutorial on Bayesian optimization of expensive cost functions, with application to active user modeling and hierarchical reinforcement learning. arXiv preprint arXiv:1012.2599. 2010.

[49] Huyer W, Neumaier A (2008). Snobfit–stable noisy optimization by branch and fit. ACM Trans Math Softw (TOMS).

[50] Huyer W, Neumaier A (1999). Global optimization by multilevel coordinate search. J Glob Optim.

[51] Rasmussen CE, Hannes Nickisch CW. GPML. http://www.gaussianprocess.org/gpml/code/matlab/doc/. v3.6-2015-07-07, Accessed 1 Aug 2016.

[52] Duvenaud D. Github - Additive Gaussian Processes. https://github.com/duvenaud/additive-gps. Accessed 30 Aug 2017.

[53] Bergstra J, Bengio Y (2012). Random search for hyper-parameter optimization. J Mach Learn Res.

[54] Sacks J, Welch W. J, Mitchell TJ, Wynn HP. Design and analysis of computer experiments. Stat Sci. 1989;409–23.

[55] Jones DR, Schonlau M, Welch WJ (1998). Efficient global optimization of expensive black-box functions. J Glob Optim.

[56] Jones DR, Perttunen CD, Stuckman BE (1993). Lipschitzian optimization without the lipschitz constant. J Optim Theory Appl.

[57] Powell MJ (2002). Uobyqa: unconstrained optimization by quadratic approximation. Math Program.

[58] Github-vcsa. https://github.com/MA-Thomas/vcsa. Accessed 24 Jan 2018.

